# Maternal diet in pregnancy and acute leukemia in infants: a case-control study in Mexico City

**DOI:** 10.3389/fonc.2023.1165323

**Published:** 2024-01-08

**Authors:** María Luisa Pérez-Saldivar, M. Karen Flores-García, Nancy Núñez-Villegas, Arturo Fajardo-Gutiérrez, Aurora Medina-Sanson, Elva Jiménez-Hernández, Jorge Alfonso Martín-Trejo, Norma López-Santiago, José Gabriel Peñaloza-González, Beatriz Cortés-Herrera, Laura Elizabeth Merino-Pasaye, Raquel Amador-Sánchez, Luis Ramiro García-López, Héctor Pérez-Lorenzana, Pedro Francisco Román-Zepeda, Alejandro Castañeda-Echevarría, María Guadalupe López-Caballero, Sofía Irene Martínez-Silva, Juan Rivera-González, Jorge Granados-Kraulles, Jesús Flores-Botello, Francisco Medrano-López, María Adriana Rodríguez-Vázquez, Delfino Torres-Valle, Karina Mora-Rico, Félix G. Mora-Ríos, Luis R.García‐Cortés, Perla Salcedo-Lozada, Janet Flores-Lujano, Juan Carlos Núñez-Enríquez, Vilma Carolina Bekker-Méndez, Minerva Mata-Rocha, Haydeé Rosas-Vargas, David Aldebarán Duarte-Rodríguez, Silvia Jiménez-Morales, Alfredo Hidalgo-Miranda, Lizbeth López-Carrillo, Juan Manuel Mejía-Aranguré

**Affiliations:** ^1^ Unidad de Investigación Médica en Epidemiología Clínica, Unidad Médica de Alta Especialidad (Unidad Médica de Alta Especialidad (UMAE)) Hospital de Pediatría, Centro Médico Nacional (Centro Médico Nacional (CMN)) Siglo XXI, Instituto Mexicano del Seguro Social (Instituto Mexicano del Seguro Social (IMSS)), Mexico City, Mexico; ^2^ Instituto Nacional de Salud Pública de México (INSP), Cuernavaca, Morelos, Mexico; ^3^ Servicio de Hematología Pediátrica, Hospital General “Gaudencio González Garza”, Centro Médico Nacional (CMN) “La Raza”, Instituto Mexicano del Seguro Social (IMSS), Mexico City, Mexico; ^4^ Departamento de Hemato-Oncología, Hospital Infantil de México Federico Gómez, Secretaria de Salud (Secretaría de Salud (SSA)), Mexico City, Mexico; ^5^ Servicio de Oncología, Hospital Pediátrico Moctezuma, Secretaría de Salud de la Ciudad de México (Secretaría de Salud de la Ciudad de México (SSCDMX)), Mexico City, Mexico; ^6^ Servicio de Hematología, Unidad Médica de Alta Especialidad (UMAE) Hospital de Pediatría, Centro Médico Nacional (CMN) “Siglo XXI”, Instituto Mexicano del Seguro Social (IMSS), Mexico City, Mexico; ^7^ Servicio de Hematología, Instituto Nacional de Pediatría (INP), Secretaría de Salud (SSA), Mexico City, Mexico; ^8^ Servicio de Onco-Pediatría, Hospital Juárez de México, Secretaría de Salud (SSA), Mexico City, Mexico; ^9^ Servicio de Hematología Pediátrica, Hospital General de México, Secretaría de Salud (SSA), Mexico City, Mexico; ^10^ Servicio de Hematología Pediátrica, Centro Médico Nacional (CMN)”20 de Noviembre”, Instituto de Seguridad Social al Servicio de los Trabajadores del Estado (Instituto de Seguridad Social al Servicio de los Trabajadores del Estado (ISSSTE)), Mexico City, Mexico; ^11^ Servicio de Hematología Pediátrica, HGR No. 1 “Dr. Carlos Mac Gregor Sánchez Navarro” Instituto Mexicano del Seguro Social (IMSS), Mexico City, Mexico; ^12^ Servicio de Pediatría, Hospital Pediátrico de Tacubaya, Secretaría de Salud de la Ciudad de México (SSCDMX), Mexico City, Mexico; ^13^ Servicio de Cirugía Pediátrica, Hospital General “Gaudencio González Garza”, Centro Médico Nacional (CMN) “La Raza”, Instituto Mexicano del Seguro Social (IMSS), Mexico City, Mexico; ^14^ Servicio de Cirugía Pediátrica, Hospital General Regional (HGR) No. 1 “Dr. Carlos Mac Gregor Sánchez Navarro” Instituto Mexicano del Seguro Social (IMSS), Mexico City, Mexico; ^15^ Servicio de Pediatría, Hospital General de Zona Regional (HGZR) No. 25 Instituto Mexicano del Seguro Social (IMSS), Mexico City, Mexico; ^16^ Coordinación Clínica y Pediatría, Hospital Pediátrico de Coyoacán, Secretaría de Salud de la Ciudad de México (SSCDMX), Mexico City, Mexico; ^17^ Hospital Pediátrico de Iztapalapa, Secretaría de Salud de la Ciudad de México (SSCDMX), Mexico City, Mexico; ^18^ Hospital General Dr. “Gustavo Baz Prada”, Instituto de Salud del Estado de México (ISEM), Estado de México, Mexico; ^19^ Coordinación Clínica y Pediatría del Hospital General de Zona 76 Instituto Mexicano del Seguro Social (IMSS), Estado de México, Mexico; ^20^ Coordinación Clínica y Pediatría, Hospital General “La Perla” ISEM, Estado de México, Mexico; ^21^ Coordinación Clínica y Pediatría, HGR No. 72 “Dr. Vicente Santos Guajardo”, Instituto Mexicano del Seguro Social (IMSS), Estado de México, Mexico; ^22^ Coordinación Clínica y Pediatría del Hospital General de Zona 68, Instituto Mexicano del Seguro Social (IMSS), Estado de México, Mexico; ^23^ Coordinación Clínica y Pediatría del Hospital General de Zona 71, Instituto Mexicano del Seguro Social (IMSS), Estado de México, Mexico; ^24^ Servicio de Cirugía Pediátrica, HGR 1° Octubre, Instituto de Seguridad Social al Servicio de los Trabajadores del Estado (ISSSTE), Mexico City, Mexico; ^25^ Cirugía Pediátrica del Hospital Regional “General Ignacio Zaragoza”, Instituto de Seguridad Social al Servicio de los Trabajadores del Estado (ISSSTE), Mexico City, Mexico; ^26^ Delegación Regional Estado de México Oriente, Instituto Mexicano del Seguro Social (IMSS), Estado de México, Mexico; ^27^ Hospital General de Ecatepec “Las Américas”, ISEM, Estado de México, Mexico; ^28^ Unidad de Investigación Médica en Inmunología e Infectología, Hospital de Infectología “Dr. Daniel Méndez Hernández”, Centro Médico Nacional (CMN) “La Raza”, Instituto Mexicano del Seguro Social (IMSS), Mexico City, Mexico; ^29^ Laboratorio de Biología Molecular de las Leucemias, Unidad de Investigación en Genética Humana, Unidad Médica de Alta Especialidad (UMAE), Hospital de Pediatría, Centro Médico Nacional (CMN) “Siglo XXI”, Instituto Mexicano del Seguro Social (IMSS), Mexico City, Mexico; ^30^ Laboratorio de Genética, Unidad Médica de Alta Especialidad (Unidad Médica de Alta Especialidad (UMAE)) Hospital de Pediatría, Centro Médico Nacional (Centro Médico Nacional (CMN)) Siglo XXI, Instituto Mexicano del Seguro Social (IMSS), Mexico City, Mexico; ^31^ Laboratorio de Innovación y Medicina de Precisión, Núcleo A. Instituto Nacional de Medicina Genómica (INMEGEN), Mexico City, Mexico; ^32^ Facultad de Medicina, Universidad Nacional Autónoma de México (UNAM), Mexico City, Mexico; ^33^ Laboratorio Genómica Funcional del Cáncer, Instituto Nacional de Medicina Genómica (INMEGEN), Mexico City, Mexico

**Keywords:** maternal diet, acute leukemia, infant leukemia, case-control, food groups, allium vegetables, high-fat dairy products, topoisomerase II

## Abstract

**Introduction:**

Epidemiological studies around the world on acute leukemia (AL) and risk factors in infants are scarce. Infant AL has been proposed to originate *in utero*, which facilitates its study by establishing a short exposure time in pregnant women to environmental and dietary factors that could contribute to the risk of or protection against leukemia. We hypothesized that maternal diet during pregnancy may be an important factor involved in AL in offspring.

**Methods:**

We conducted a hospital-based case-control study from 2010 to 2019 on maternal diet during pregnancy in nine high-specialty public hospitals of different health institutions that diagnose and offer treatment to children with AL in Mexico City. Cases (n=109) were children ≤24 months of age with *de novo* diagnosis of AL, and controls (n=252) were children obtained in hospitals from second-level medical care matched for age, sex, and health institution. Maternal diet during pregnancy was obtained by a semiquantitative food frequency questionnaire. Unconditional logistic regression models were used to assess the association between food groups and infant AL. Potential confounders were assessed by constructing directed acyclic graphs (DAGs) with Dagitty software in which adjusted options were identified for the construction of unconditional logistic regression models.

**Results:**

Cases were slightly predominantly female (52.3%). The years of education of the mother in cases and controls was 0-9 on average, and those who reported smoking cigarettes and consuming alcohol during pregnancy did so at a low frequency. Regarding the mother’s diet, the main findings were that the consumption of allium vegetables during pregnancy was inversely associated with AL for medium and high consumption (OR=0.26, 95% CI 0.14-0.46; *P*-trend< 0.001). In contrast, the high consumption of high-fat dairy products had a positive association with AL (OR=2.37, 95% CI 1.30-4.34; *P*-trend<0.001). No association was found between consumption of topoisomerase II inhibitor foods during pregnancy and AL.

**Conclusion:**

The results suggest that maternal intake during pregnancy of allium vegetables, specifically garlic, is inversely associated with the development of AL in children ≤24 months old. On the other hand, consumption of high-fat dairy products is positively associated with AL in children ≤24 months old.

## 1 Introduction

Leukemia is the most common childhood malignancy worldwide (1). Acute leukemias (AL) are the most frequent among all leukemias in children, the most important subgroups among them being acute lymphoblastic leukemia (ALL) and acute myeloid leukemia (AML). ALL is the most frequent type of leukemia constituting 80%, followed by AML with a little more than 10%, together ALL and AML comprise about 30%-35% of all cancers diagnosed in children under 15 years of age (1, 2). Leukemia occurring in the first 12 months of age is rare, nevertheless it is the second most frequent type of cancer in children under one year of age, and distinct from leukemia in older children by clinical, biological and epidemiological characteristics, for example, ALL in children under 1 year of age are considered to be at high risk for relapse or death (3, 4). Another important aspect to highlight in infants with acute leukemia (AL) is the survival compared to older children once they start the treatment protocol, reporting a very low 5-year survival rate (~50% vs. 67-87%) (5).

To date there is convincing evidence that infant leukemia originates *in utero* before birth, demonstrated by analysis of neonatal blood spots or Guthrie cards, in which the same sequence of fusion genes has been observed in a neonatal blood spot as in the patient’s leukemic cells at the time of diagnosis. In addition, studies performed in twins and analysis of the Guthrie cards corresponding to those twins provides unequivocal proof that acute leukemia is of fetal origin in all infants (6).

Maternal diet plays an integral role in shaping newborn body composition, genome stability and immune system development. Thus, maternal diet is crucial for fetal development and predisposition to disease (7). In addition, the study of AL in infants and maternal diet during pregnancy is absolutely necessary because they have a very short latency period, which represents a unique opportunity in the etiology of the disease.

Epidemiological studies on maternal diet and AL have focused mainly on school-age children, identifying some foods, beverages and supplements associated with the development of AL in children, and there is even consistency in the results with which it has been possible to recommend a balanced diet during pregnancy. The results of these studies support that the consumption of vegetables, fruits, seafood, tea, folate supplementation, vitamins A and D during pregnancy decrease the risk of AL in their children (8–10). Conversely, the consumption of smoked meats, red meats, sugars, sweet syrups, coffee, cola and alcohol during pregnancy increases the risk of AL in their children (11–15).

The study of maternal diet in infants has been little studied, epidemiological studies have focused on the diet with foods that inhibit the function of the enzyme DNA Topoisomerase II, as it has been reported that infants with AL have a chromosomal aberration called *KMT2A-r* (also known as MLL -mixed-lineage or myeloid-lymphoid leukemia- gene at 11q23), at a frequency of 31% to 79% (16). This enzyme is responsible for unwinding and rebinding DNA during cell division (3). *KMT2A-r* was identified as a secondary outcome of treatment of AL in adults with drugs such as epipodophyllotoxins, which are inhibitors of the enzyme DNA topoisomerase II (17). However, it was necessary to know whether maternal diet during pregnancy with inhibitors of the enzyme DNA Topoisomerase II, contained in some foods naturally, could be a risk for the development of AL in their children. The list of foods included legumes, onions, apples, soy products, coffee and other caffeinated beverages such as black tea, green tea, cocoa and red wine (18). They found a significant positive association with increased consumption of foods containing enzyme DNA Topoisomerase II inhibitors for medium and high consumption during pregnancy and risk of AML in infants; for ALL, they found no significant risk or trend. Subsequently, the Children Oncology Group (COG) continued this study with a greater number of infant cases and controls, finding an association between consumption of foods containing enzyme DNA Topoisomerase II inhibitors and risk of AML with *KMT2A-r* (+). Also, they observed a trend in risk with increasing consumption of enzyme DNA Topoisomerase II inhibitor (19). Afterwards, Petridou et al. conducted a study on maternal diet during pregnancy and its association with ALL in young children aged 12 to 59 months. They found a risk of ALL associated with the consumption by the mother during pregnancy of sugars and syrups, meat and its derivatives, and a protective effect of the consumption of fruits and vegetables (20).

Surprisingly, the studies were not replicated in infants to test the findings that foods that inhibit the function of the enzyme DNA Topoisomerase II included in the maternal diet during pregnancy are a risk for AL in infants, nor were the studies replicated in infants to test the findings that foods that inhibit the function of the enzyme DNA Topoisomerase II included in the maternal diet during pregnancy are a risk for AL in infants, neither, have not been replicated in Hispanic population and have not been studied in the Mexican population residing in Mexico City. Therefore, a study focused on maternal diet in our population is needed to determine its role in the development of AL among in their offspring.

The aim of the present study was to evaluate maternal diet during pregnancy and its association with AL in their offspring in Mexico City.

## 2 Materials and methods

A hospital-based case-control study was conducted in Mexico City during the period 2010 to 2019. The study population consisted of mother-child pairs identified in nine high-specialty public hospitals (Centro Médico Nacional La Raza “Dr. Gaudencio González Garza” IMSS, Hospital Infantil de México “Federico Gómez” Ssa, Hospital Pediátrico de Moctezuma SS CDMX, Hospital de Pediatría “Dr. Silvestre Frenk Freund” Centro Médico Nacional Siglo XXI IMSS, Instituto Nacional de Pediatría Ssa, Hospital Juárez de México Ssa, Hospital General de México “Dr. Eduardo Liceaga” Ssa, Centro Médico Nacional “20 de Noviembre” ISSSTE, Hospital General Regional No. 1 “Dr. Carlos MacGregor Sánchez Navarro” IMSS). Patients included in this study were all the public hospitals (i.e., dependent on the government) that provide medical care for children with leukemia in Mexico City. It has been shown that these public hospitals treat 97.5% of all the cases of leukemia occurring in Mexico City (21). Controls were collected from hospitals that treat the four basic specialties: internal medicine, surgery, pediatrics, and gynecology-obstetrics of the same health institutions where the cases were diagnosed and treated. The protocol was approved by the IMSS National Committee for Scientific Research with number 2010-785-064.

Patients had to be biological children of the person identified as the mother of the child in order to apply the semi-quantitative food frequency questionnaire. This would allow us to know what foods were consumed during pregnancy. The other criterion was their residence in Mexico City and in the State of Mexico, because although these hospitals receive some pediatric patients from other states in the interior of the country, it would not be possible to obtain information from control children from the same states as the cases and also the diet varies by state of the Mexican Republic, so the way to use a standard instrument is that it could clearly delimit the area where both the case children and the control children came from. On the other hand, if the parents of the children refused to participate in the study, these patients were excluded from the study.

### 2.1 Cases

Cases were children ≤ 24 months old with a diagnosis of *de novo* AL confirmed by bone marrow smear, histochemical tests (myeloperoxidase, Sudan black B reaction, esterases, periodic Schiff reaction [PAS], and acid phosphatase), and immunophenotyping. A total of 250 patients were identified using the following eligibility criteria: age range between 0 and 24 months, residents of Mexico City and the State of Mexico, confirmed and recent diagnosis of AL treated in one of the nine high-specialty public hospitals, and living with their biological mother who had agreed to participate in the study. Of the 250 eligible pairs, 13 were excluded because the child had Down syndrome, 127 pairs could not be given the diet questionnaire (11 patients were diagnosed in 2010 when the instrument was not yet available and could not be recovered, 11 patients whose mothers did not agree to participate, 10 patients who were treated in an institution that did not participate in the study, 55 patients who died within 2 months of diagnosis and could not be interviewed, 2 patients who were lost due to the COVID-19 pandemic, and 38 patients who were lost to follow-up and were not administered the maternal diet questionnaire), leaving 110 pairs participating. Thus, the participation rate of the study was 44.0% (**Annex 1**).

### 2.2 Controls

The children who were selected as controls were those younger than 36 months and who were selected from the same health institution from which the cases emerged at second-level hospitals. The Mexican public health system is structured in three levels of care: first level or primary units that provide medical care mainly by family physicians; if patients require care from specialist physicians, they are sent to the second level where four basic specialties are concentrated (internal medicine, surgery, pediatrics, and gynecology-obstetrics). In the third level of public health care in our country, the most complex diseases are treated, which require specialized equipment and facilities, such as cancer, cardiac, orthopedic, metabolic, cardiovascular, renal, ophthalmologic, neurological, rheumatologic diseases, etc. (22). Controls were recruited at second-level hospitals from ambulatory surgery services (e.g., for circumcision, hernioplasty, orchidopexy, tonsillectomy) in short-stay facilities and in the emergency department (e.g., for trauma, intoxication, first and second level burns). The selection criteria were living with their biological mother who agreed to participate in the study. In total, 277 eligible controls were identified, 24 of whom did not accept to participate, leaving 253 participating pairs, a participation rate of 91.3% (**Annex 1**).

### 2.3 Interviews

Prior to the interview, informed consent to participate was obtained from the parents. The mothers of both cases and controls were interviewed directly in the hospitals by trained personnel to obtain information on sociodemographic variables, child birth, parental alcohol and tobacco consumption, and maternal diet during pregnancy.

### 2.4 Maternal diet

Mothers were interviewed about the consumption of 109 foods and beverages, as well as 7 dishes during the pregnancy of the recruited child, using a semi-quantitative food frequency questionnaire (FFQ). The reproducibility of this instrument was evaluated in Mexican women, to whom the questionnaire was administered twice in a 1-year interval, whereas its validity was estimated using 24-hour reminders at 3-month intervals as a reference (23).

According to the methodology suggested by Willett et al. (24), the FFQ includes 10 frequency of consumption response options ranging from “never” to “6 or more times a day”, as well as predetermined portions for each food as follows: one glass (for milk and wine), one cup (for yogurt, some fruits and vegetables, tea, juices, alcoholic beverages and soft drinks), one spoon (for oils, sour cream, sauces and nuts), one slice (for cheeses, some fruits and meats), one plate (for legumes and local dishes), and one piece (for some fruits and breads).

A database was created with the nutritional and energy content of each food obtained from the nutritional composition tables of the United States Department of Agriculture (25), which include a wide variety of foods consumed in Mexico. For local foods not included in these tables, such as tejocote, reference tables from the Instituto Nacional de Ciencias Médicas y Nutrición Salvador Zubirán were used (26). Two foods were not found in any of the tables (soy juice and soy beer) and, therefore, were not included in the subsequent calculation of total energy intake.

The mothers’ total daily kilocalorie intake was calculated by adding the kilocalories contributed by the respective foods, beverages, and dishes reported during pregnancy. Because some fruits and vegetables are only consumed during some seasons of the year, their energy intake was adjusted according to their availability; for example, only 50% of the kilocalories of plums were considered because they are available only during 6 months of the year (27). A serving, or serving size, is the amount of food listed on a product’s Nutrition Facts label, or food label. Different products have different serving sizes. Sizes can be measured in cups, ounces, grams, pieces, slices, or numbers. Depending on how much you choose to eat, your serving size may or may not match the serving size. To find out how many servings a container holds, look at the top of the label. “Servings per container” appears just above “Serving Size” (28).

In this phase of the study, one case was eliminated because the total estimated energy was less than 525 kcal, which corresponds to ± 2 standard deviations of the daily intake observed in a study with pregnant Mexican women (29). Therefore, the final sample size was 109 cases and 252 controls.

### 2.5 Food groups

The foods and beverages contained in the FFQ were categorized into 28 food groups taking into account the similarity in macronutrient and micronutrient content (e.g., fat, carbohydrates, protein, vitamins, sodium), added sugar content (e.g., dairy with added sugar, cereals with fat and added sugar), and type of fat (saturated or vegetable). Some of the individual foods were considered as groups by themselves because their nutritional content did not meet the criteria for belonging to any particular group (e.g., egg, chicken, blueberries) or because of their high consumption among the population (e.g., corn tortilla and corn). In addition, a group was formed of Mexican dishes and another group of foods previously identified as inhibitors of the enzyme DNA topoisomerase II (apples, strawberries, dried blueberries, onion, beans, soy milk, soy cheese, soy meat, red wine, soda, energy drinks, coffee, black tea, green tea) (18, 19).

Sociodemographic variables were categorized based on previous studies (30, 31): sex (male, female), child’s age (≤12 months, >12-24 months), mother’s age at pregnancy (< 35 years, ≥35 years), birth weight (≤3500 grams, >3500 grams) (32), breastfeeding (≤6 months, >6 months), and parents’ years of schooling (0-9 years, 9.1-12.9 years, and ≥13 years). In addition, they were asked about habits such as parental smoking during pregnancy (yes, no) and alcohol consumption during pregnancy (yes, no). Socioeconomic status was analyzed using the mother’s years of education as in studies such as those of the Childhood Leukemia International Consortium (CLIC) (33, 34). The number of persons/bedroom in the dwelling (the overcrowding index was included as an initial variable for socioeconomic level) was also considered (low socioeconomic level ≥1.6 persons, moderate and high <1.6 persons) (35).

### 2.6 Statistical analysis

Median consumption of food groups and total energy were compared between cases and their respective controls using the Mann-Whitney U test. Non-conditional logistic regression models were used to assess the association between tertiles of consumption of each food group, (according to the distribution of consumption, is per day observed in the control group) and childhood leukemia. Potential confounders were assessed by constructing directed acyclic graphs (DAGs) (36) with Dagitty software (version 3.0) and two adjustment options were identified. The criteria established in epidemiology that a confounding variable must meet were considered in the DAG (37). The DAG was included in **Annex 2**. The variables required in model A were mother’s age, state of residence, person/bedroom ratio, breastfeeding, supplement consumption, tobacco, and alcohol. The adjustment variables in model B were mother’s years of education, state of residence, supplement consumption, alcohol, and tobacco. Model B was selected. Trend was assessed by logistic regression analysis using food groups as a continuous variable in the models. Results with a *P*-value <0.05 were considered significant.

## 3 Results

A total of 109 cases and 252 controls participated in this study (**Table 1**). Among the cases, the female sex predominated (52.3%), the age at diagnosis of AL was mostly between 12 and 24 months of life (68.8%), with a lower proportion among those younger than 12 months of life (31.2%). In relation to birth weight and having been breastfed, no differences were observed between the groups, except when breastfeeding older than 6 months was evaluated, as the frequency was higher among the controls. Regarding maternal characteristics, most of the mothers in both cases and controls were under 35 years of age at the time of pregnancy. The years of maternal education among cases was up to 9 years of schooling (51.4%), whereas mothers of controls had slightly more years of education on average (9 to 12 years of education). We found that the frequency of smoking in both case and control mothers was higher one year before pregnancy than the frequency reported during pregnancy; they decreased their cigarette consumption during pregnancy, however, although they decrease their cigarette smoking, there are women who continue to smoke even during pregnancy (3.7% and 2.8%). Notably, the mothers of the cases reported less alcohol consumption during pregnancy, whereas the mothers of the controls reported more alcohol consumption at this stage (3.7% vs. 12.3%; OR = 0.27, 95% CI 0.09-0.79). A slightly greater percentage was observed among the mothers of the controls who took iron vitamins and minerals during their pregnancy compared to the mothers of the cases. Regarding the characteristics of the parents of cases and controls, on average they reported an age <35 years at the time of pregnancy, and the level of education was very similar between the parents of cases and controls, being 9 years of study on average. Parents of cases reported slightly less alcohol and tobacco use 1 year before pregnancy compared to parents of controls (**Table 1**).

**Table 1:** Characteristics of study participants.

Variable	Groups	Cases 109	%	Control 252	%	OR^a^	95% CI	P-value
Child´s sex	Female	57	52.3	100	39.7	1.66	1.05-2.62	0.026
	Male	52	47.7	152	60.3			
Child´s age (months) at diagnosis/recruitment	≤ 12	34	31.2	73	29.0	1.11	0.68-1.81	0.671
	>12^a^	75	68.8	179	71.0			
Birth weight (grams)	≤3,500^a^	92	84.4	217	86.1			
	>3,500	17	15.6	35	13.9	1.14	0.61-2.14	0.671
Breastfeeding	Yes	96	88.1	226	89.7	0.84	0.42-1.72	0.651
	No	13	11.9	26	10.3			
Breastfeeding duration (months)	≤6	55	57.3	99	43.8	0.58	0.35-0.94	0.026
	>6	41	42.7	127	56.2			
Maternal age at pregnancy (years)	<35	97	89.0	231	91.7			
	≥35	12	11.0	21	8.3	1.36	0.64-2.87	0.417
Mother´s education (years)	0-9	56	51.4	120	47.6	1.30	0.79-2.16	0.301
	9.1-12.9^a^	34	31.2	95	37.7	*Ref.*		
	≥13	19	17.4	37	14.7	1.43	0.73-2.83	0.295
Smoking 1 year prior to pregnancy	Yes	31	28.4	77	30.6	0.90	0.55-1.48	0.686
	No	78	71.6	175	69.4			
Smoking during pregnancy	Yes	4	3.7	7	2.8	1.33	0.38-4.65	0.650
	No	105	96.3	245	97.2			
Alcohol during pregnancy	Yes	4	3.7	31	12.3	0.27	0.09-0.79	0.010
	No	105	96.3	221	87.7			
Iron during pregnancy	Yes	76	69.7	216	85.7	0.38	0.22-0.66	0.000
	No	33	30.3	36	14.3			
Vitamins during pregnancy	Yes	98	89.9	238	94.4	0.52	0.23-1.19	0.119
	No	11	10.1	14	5.6			
Minerals during pregnancy	Yes	4	3.7	16	6.3	0.56	0.18-1.72	0.307
	No	105	96.3	236	93.7			
Drug use for genital infection during pregnancy	Yes	15	13.8	34	13.5	1.02	0.53-1.97	0.945
	No	94	86.2	218	86.5			
Paternal age at pregnancy (years)	<35	82	75.2	198	78.6			
	≥35	27	24.8	54	21.4	1.21	0.71-2.05	0.488
Father´s education (years)	0-9	53	48.6	138	54.7	0.82	0.49-1.37	0.454
	9.1-12.9^a^	35	32.1	75	29.8	*Ref.*		
	≥13	21	19.3	39	15.5	1.15	0.59-2.24	0.673
Smoking 1 year prior to pregnancy	Yes	48	47.1	141	58.0	0.64	0.40-1.02	0.061
	No	61	52.9	111	42.0			
Place of residence	Mexico City	42	38.5	132	52.4	0.57	0.36-0.90	0.016
	State of Mexico	67	61.5	120	47.6			
Health institution	Ministry of health*	62	56.9	164	65.1	1.41	0.89-2.24	0.139
	IMSS^¥^	47	43.1	88	34.9			
Overcrowding index ‡	Low SES (≥1.6)	15	13.8	23	9.1	1.59	0.79-3.18	0.188
	Moderate-High SES (<1.6)^a^	94	86.2	229	90.9			

^a^ OR crude.

a Reference level. The selection was based on the CLIC analysis, and schooling <9 years was considered the high-risk or low socioeconomic level category. If a mother had a level of schooling higher than high school, it was considered that it could potentially have a protective effect, so these strata were left out and the intermediate group was considered as the reference value.

*Includes Secretary of Health, Secretary of Health of Mexico City, State of Mexico Institute of Health (ISEM, by its acronym in Spanish), Institute of Security and Social Services for State Workers (ISSSTE, by its acronym in Spanish).

¥ Mexican Institute of Social Security.

‡ It was calculated by number of persons per bedroom as socioeconomic level.

The dietary characteristics of the women during pregnancy are shown in **Table 2**, which reports the consumption of food groups by percentile, with the average consumption at the 50th percentile and the minimum and maximum consumption at the 10th and 90th percentile, respectively. Significant differences were observed between cases and controls in the consumption of some foods, such as high-fat dairy products, saturated fats, allium vegetables, corn, alcohol drinks, avocado, and dishes (*P*<0.05). The most striking food group in this study was allium vegetables, which were consumed at higher levels during pregnancy among mothers of controls than among mothers of cases (*P*=0.0001).

**Table 2:** Dietary characteristics of the study sample (Energy (kcal/day).

Energy (kcal/day)		All participants	Cases	Controls	*P*-value
		p50 (p10, p90)			
		2191.14 (1413.11, 3305.08)	2271.09 (1402.20, 3425.84)	2159.71 (1413.105, 3204.94)	0.367
Food group	Food items				
High-fat dairy products	Milk, Oaxaca cheese, fresh cheese, Manchego	1.00 (0.29, 2.46)	1.23 (0.35, 3.06)	0.87 (0.29, 1.86)	0.0002*
Dairy with added sugar	Ice cream, yogurt	0.29 (0.03, 1.07)	0.21 (0.02, 1.14)	0.29 (0.03, 1.02)	0.7803
Citrus fruits	Oranges, orange juice, tangerines, tangerine juice, grapefruit, grapefruit juice	0.64 (0.14, 2.02)	0.53 (0.14, 1.82)	0.71 (0.14, 2.11)	0.0917
Other fruits	Bananas, peaches, grapes, melon, watermelon, mango, pears, cactus fruit, papaya, pineapple, plums, blackberries, mamey, zapote	1.31 (0.49, 3.09)	1.21 (0.49, 3.14)	1.42 (0.49, 3.10)	0.0844
Egg	Egg	0.86 (0.13, 1.29)	0.86 (0.14, 1.57)	0.79 (0.13, 1.29)	0.2326
Chicken	Chicken	0.43 (0.14, 0.43)	0.43 (0.14, 0.79)	0.43 (0.14, 0.43)	0.4847
Processed meats	Sausage, ham, chorizo, bacon	0.49 (0.13, 1.21)	0.51 (0.13, 1.29)	0.49 (0.13, 1.21)	0.6059
Red meat	Beef, pork, cecina, barbecue, carnitas, liver	0.45 (0.12, 0.99)	0.48 (0.14, 1.01)	0.43 (0.08, 0.92)	0.1278
Fish and shellfish	Tuna, fish, shellfish, sardines	0.20 (0.03, 0.58)	0.16 (0.03, 0.57)	0.20 (0.03, 0.64)	0.1853
Saturated fats	Pork rinds, cream, butter, mayonnaise, lard	0.64 (0.16, 1.43)	0.45 (0.14, 1.57)	0.70 (0.18, 1.43)	0.0042*
Cruciferous vegetables	Broccoli, cauliflower, cabbage	0.27 (0, 0.92)	0.29 (0.00, 0.92)	0.21 (0.00, 0.92)	0.6769
Allium vegetables	Garlic	0.79 (0.00, 1.00)	0.43 (0.00, 1.00)	0.79 (0.07, 1.00)	0.0001*
Green leafy vegetables	Verdolagas, spinach, lettuce, parsley	0.46 (0.14, 1.43)	0.45 (0.14, 1.16)	0.49 (0.13, 1.56)	0.2362
Other vegetables	Squash, chayote, stewed tomato, raw tomato, nopal, chili, squash flower	2.19 (1.04, 3.93)	2.15 (1.04, 4.58)	2.19 (1.10, 3.67)	0.8159
Corn	Corn	0.14 (0.00, 0.43)	0.07 (0.00, 0.43)	0.14 (0.00, 0.43)	0.0154
Legumes	Lentils, broad beans, peas	0.21 (0.02, 0.86)	0.15 (0.02, 0.71)	0.21 (0.02, 0.86)	0.1176
Corn tortilla	Corn tortilla	4.00 (1.29, 10.00)	4.71 (1.00, 12.50)	4.00 (1.57, 10.00)	0.3818
Cereals high in fat and sugar	Cookies, cake, sweet bread	0.51 (0.13, 1.13)	0.51 (0.08, 1.29)	0.51 (0.13, 1.08)	0.7229
Alcoholic drinks	White wine, beer, spirits	0.00 (0.00, 0.08)	0.00 (0.00, 0.03)	0.00 (0.00, 0.13)	0.0159
Tea	Herbal tea	0.00 (0.00, 0.43)	0.02 (0.00, 0.43)	0.00 (0.00, 0.43)	0.3579
Atole	Atole	0.07 (0.00, 0.43)	0.07 (0.00, 0.43)	0.14 (0.00, 0.43)	0.5533
Avocado	Avocado	0.14 (0.00, 0.43)	0.14 (0.00, 0.43)	0.14 (0.00, 0.79)	0.0121
Mexican dishes	Pastor, quesadilla, pozole, gordita, pambazo, tamal, sope	0.46 (0.11, 1.12)	0.40 (0.03, 1.13)	0.46 (0.16, 1.10)	0.0139
DNA topoisomerase II enzyme inhibitors	Apples, strawberries, dried cranberries, onion, beans, soy milk, soy cheese, soy meat, soy sauce, red wine, soft drinks, energy drinks, coffee, green tea, black tea	2.57 (1.31, 4.41)	2.49 (1.01, 4.61)	2.60 (1.45, 4.21)	0.1555

p50 (p10, p90) = Values are given as 50th percentile outside the parentheses and inside the parentheses 10th percentile and 90th percentile.

**Table 3** presents the results of the logistic regression analysis adjusted for the variables obtained by the DAGs. The two models were model “A”, adjusted for mother’s age, state of residence, person/habitation ratio, breastfeeding, consumption of food supplements, and tobacco and alcohol consumption, and model “B”, adjusted for mother’s education, state of residence, dietary supplement consumption, smoking, and alcohol consumption. Both models had very similar results.

**Table 3:** Association between food groups and acute leukemia.

Food group (servings/day)	(Ca/Co)	OR (95% CI)^a^	*P*-trend	OR (95% CI)^b^	*P*-trend	OR (95% CI)^c^	*P*-trend
High-fat dairy products							
<0.5716	(23/83)	1.00		1.00		1.00	
<0.5716-<1.2143	(30/81)	1.34 (0.72, 2.49)	**<0.001**	1.34 (0.69, 2.60)	**<0.001**	1.35 (0.70, 2.60)	**<0.001**
≥1.2143	(56/87)	**2.32 (1.31, 4.11)**		**2.29 (1.25, 4.21)**		**2.37 (1.30, 4.34)**	
Dairy with added sugar							
<0.1593	(47/88)	1.00		1.00		1.00	
≥0.1593 - <0.4944	(23/76)	0.57 (0.32, 1.02)	0.786	0.49 (0.26, 0.91)	0.417	0.50 (0.27, 0.94)	0.441
≥0.4944	(39/87)	0.84 (0.50, 1.41)		0.76 (0.43, 1.34)		0.77 (0.44, 1.33)	
Citrus fruits							
<0.533	(39/84)	1.00		1.00		1.00	
≥0.533, < 1.0546	(46/83)	0.40 (0.22, 0.73)	0.366	0.44 (0.24, 0.82)	0.237	0.43 (0.23, 0.81)	0.217
≥1.0546	(24/85)	0.61 (0.36, 1.04)		0.58 (0.32, 1.01)		**0.56 (0.32, 0.98)**	
Other fruits							
< 1.000625	(46/85)	1.00		1.00		1.00	
≥ 1.000625- <1.980433	(39/82)	1.07 (0.59, 1.94)	0.262	1.21 (0.50, 2.10)	0.139	1.17 (0.67, 2.03)	0.14
≥1.980433	(24/85)	0.62 (0.34, 1.13)		0.57 (0.31, 1.06)		0.57 (0.31, 1.06)	
Egg							
<0.2858	(18/48)	1.00		1.00		1.00	
≥0.2858-<0.8572	(27/76)	0.95 (0.47, 1.90)	0.956	1.01 (0.48, 2.13)	0.932	1.00 (0.48, 2.09)	0.958
≥0.8572	(62/123)	1.34 (0.72, 2.50)		1.48 (0.76, 2.87)		1.46 (0.76, 2.81)	
Poultry							
≤0.4286	(30/76)	1.00		1.00		1.00	
<0.4286-≤0.7857	(66/154)	1.09 (0.65, 1.81)	0.327	1.08 (0.62, 1.87)	0.728	1.10 (0.64, 1.89)	0.684
>0.7857	(13/22)	1.50 (0.67, 3.35)		1.37 (0.58, 3.25)		1.40 (0.60, 3.27)	
Processed meats							
<0.3516	(31/83)	1.00		1.00		1.00	
≥0.3516 -<0.8515	(44/84)	1.40 (0.81, 2.43)	0.826	1.33 (0.74, 2.38)	0.715	1.37 (0.77, 2.44)	0.719
≥0.8515	(34/85)	1.07 (0.60, 1.90)		1.06 (0.58, 1.94)		1.05 (0.58, 1.92)	
Red meat							
<0.296	(31/83)	1.00		1.00		1.00	
≥0.296 -<0.5715	(31/80)	1.04 (0.58, 1.86)	0.336	1.27 (0.68, 2.37)	0.225	1.20 (0.65, 2.24)	0.205
≥0.5715	(47/88)	1.43 (0.83, 2.46)		1.51 (0.84, 2.70)		1.48 (0.83, 2.64)	
Fish and shellfish							
<0.1429	(41/79)	1.00		1.00		1.00	
≥0.1429 -<0.2909	(39/88)	0.85 (0.50, 1.46)	0.069	0.91 (0.52, 1.60)	0.059	0.91 (0.52, 1.59)	0.073
≥0.2909	(29/85)	0.66 (0.37, 1.16)		0.58 (0.31, 1.07)		0.62 (0.34, 1.14)	
Rich in saturated fats							
<0.4945	(62/79)	1.00		1.00		1.00	
≥0.4945-<0.9394	(23/88)	0.33 (0.19, 0.59)	0.062	0.36 (0.20, 0.65)	0.151	0.35 (0.19, 0.63)	0.123
≥0.9394	(23/84)	**0.35 (0.20, 0.62)**		**0.39 (0.21, 0.72)**		**0.38 (0.20, 0.69)**	
Cruciferous vegetables							
<0.1316	(31/74)	1.00		1.00		1.00	
≥0.1316 - 0.4286	(33/84)	0.94 (0.52, 1.68)	0.828	1.02 (0.54, 1.93)	0.850	1.07 (0.58, 1.97)	0.820
≥0.4286	(45/94)	1.14 (0.66, 1.98)		1.10 (0.61, 1.99)		1.11 (0.62, 1.98)	
Allium vegetables							
<0.7857	(57/53)	1.00		1.00		1.00	
≥0.7857 -<1	(17/102)	0.54 (0.25, 1.20)	**0.004**	0.15 (0.08, 0.30)	**<0.001**	0.15 (0.08, 0.30)	**<0.001**
≥1	(34/97)	**0.26 (0.15, 0.46)**		**0.27 (0.15, 0.49)**		**0.26 (0.14, 0.46)**	
Green leafy vegetables							
<0.3403	(37/82)	1.00		1.00		1.00	
≥0.3403 -<0.7144001	(49/86)	1.27 (0.75, 2.13)	0.047	1.38 (0.78, 2.44)	0.042	1.30 (0.74, 2.28)	0.037
≥0.7144001	(23/84)	0.61 (0.33, 1.11)		0.58 (0.30, 1.10)		0.57 (0.30, 1.08)	
Other vegetables							
< 1.84478	(47/84)	1.00		1.00		1.00	
≥ 1.84478-<2.679343	(19/83)	0.41 (0.22, 0.76)	0.357	0.47 (0.35, 0.90)	0.415	0.46 (0.24, 0.86)	0.523
≥2.679343	(42/84)	0.89 (0.53, 1.49)		0.89 (0.51, 1.54)		0.85 (0.50,1.47)	
Corn							
<0.1429	(66/112)	1.00		1.00		1.00	
≥0.1429-<0.4286	(30/74)	0.69 (0.41, 1.16)	**0.01**	0.75 (0.43, 1.30)	**0.012**	0.73 (0.42, 1.27)	**0.014**
≥0.4286	(13/66)	**0.33 (0.17, 0.65)**		**0.31 (0.15, 0.62)**		**0.32 (0.16, 0.64)**	
Root vegetables							
<0.3516	(33/84)	1.00		1.00		1.00	
≥0.3516 -<0.8572	(27/53)	1.30 (0.70, 2.40)	0.603	1.10 (0.56, 2.14)	0.500	1.12 (0.58, 2.17)	0.592
≥0.8572	(48/115)	1.06 (0.63, 1. 80)		1.07 (0.62, 1. 86)		1.10 (0.63, 1.90)	
Legumes							
<0.1429	(38/74)	1.00		1.00		1.00	
≥0.1429 -<0.3516	(40/91)	1.57 (0.55, 4.50)	0.102	0.94 (0.53, 1.68)	0.373	0.97 (0.55, 1.72)	0.323
≥0.3516	(31/87)	1.16 (0.44, 3.06)		0.79 (0.43, 1.43)		0.76 (0.42, 1.37)	
Canned chili peppers							
≤0.0164	(29/68)	1.00		1.00		1.00	
>0.0164-<0.1429	(34/50)	1.59 (0.86, 2.95)	0.303	1.77 (0.91, 3.44)	0.300	1.60 (0.83, 3.08)	0.261
≥0.1429	(46/134)	0.81 (0.46, 1.39)		0.78 (0.44, 1.41)		0.74 (0.41, 1.33)	
Corn tortilla							
<3	(22/57)	1.00		1.00		1.00	
≥3-<5	(33/91)	0.94 (0.50, 1. 77)	0.391	0.96 (0.49, 1.89)	0.925	0.90 (0.46, 1.76)	0.894
≥5	(54/99)	1.41 (0.78, 2.56)		1.32 (0.70, 2.51)		1.24 (0.65, 2.37)	
Cereals							
<1.7144	(54/81)	1.00		1.00		1.00	
≥1.7144-<2.5	(22/78)	0.42 (0.24, 0.76)	0.431	0.52 (0.28, 0.96)	0.348	0.48 (0.25, 0.86)	0.253
≥2.5	(31/84)	**0.55 (0.32, 0.95)**		**0.53 (0.30, 0.94)**		**0.50 (0.28, 0.89)**	
Cereals high in fat and sugar							
<0.4286	(34/78)	1.00		1.00		1.00	
≥0.4286-<0.6373	(36/82)	1.01 (0.57, 1.77)	0.330	0.81 (0.44, 1.48)	0.958	0.86 (0.45, 1.48)	0.889
≥0.6373	(39/91)	0.78 (0.57, 1.70)		0.75 (0.42, 1.35)		0.78 (0.44, 1.41)	
Alcoholic drinks							
0	(79/158)	1.00		1.00		1.00	
>0-<0.0658	(24/42)	1.16 (0.65, 2.05)	**0.003**	1.09 (0.59, 2.00)	**0.002**	1.03 (0.56, 1.89)	**0.001**
≥0.0658	(5/52)	0.18 (0.07, 0.47)		0.17 (0.06, 0.47)		0.17 (0.06, 0.46)	
Tea							
0	(50/140)	1.00		1.00		1.00	
>0-<0.4286	(38/59)	0.93 (0.37, 2.35)	0.503	1.82 (1.04, 3.17)	0.575	1.89 (1.09, 3.26)	0.645
≥0.4286	(21/53)	0.49 (0.22, 1.12)		1.09 (0.58, 2.07)		1.13 (0.60, 2.12)	
Corn-based drinks							
<0.0658	(34/65)	1.00		1.00		1.00	
≥0.0658-<0.1429	(26/60)	0.83 (0.45, 1.54)	0.992	0.98 (0.51, 1. 89)	0.922	0.95 (0.49, 1.81)	0.826
≥0.1429	(49/127)	0.74 (0.43, 1.25)		0.81 (0.46, 1.43)		0.76 (0.44, 1.34)	
Avocado							
<0.1429	(45/76)	1.00		1.00		1.00	
≥0.1429-<0.4286	(38/80)	0.80 (0.47, 1. 37)	**0.015**	0.79 (0.45, 1. 40)	**0.053**	0.83 (0.47, 1.45)	**0.040**
≥0.4286	(26/96)	**0.46 (0.26, 0.81)**		**0.55 (0.30, 1.00)**		**0.52 (0.29, 0.95)**	
Vegetable fats							
<0.9286	(24/82)	1.00		1.00		1.00	
≥0.9286 -<1.1429	(45/85)	1.81 (1.01, 3.23)	0.208	1.73 (0.93, 3.21)	0.310	1.79 (0.97, 3.31)	0.354
≥1.1429	(38/81)	1.60 (0.88, 2.91)		1.60 (0.85, 3.03)		1.56 (0.83, 2.94)	
Mexican dishes							
<0.368	(51/83)	1.00		1.00		1.00	
≥0.368-<0.6538	(28/84)	0.54 (0.31, 0.94)	0.114	0.53 (0.30, 0.96)	0.132	0.50 (0.28, 0.89)	0.115
≥0.6538	(29/85)	**0.56 (0.32, 0.96)**		**0.55 (0.31, 0.98)**		**0.53 (0.29, 0.94)**	
DNA topoisomerase II enzyme inhibitors							
< 2.28585	(45/84)	1.00		1.00		1.00	
≥2.28585-< 3.0987	(33/83)	0.74 (0.43, 1.28)	0.212	0.63 (0.35, 1.11)	0.135	0.66 (0.37, 1.16)	0.127
≥3.0987	(31/85)	0.68 (0.39, 1.18)		0.62 (0.34, 1.11)		0.62 (0.35, 1.11)	

a crude model,

b adjusted by mothers age, state of residence, person/room ratio (overcrowding level), breastfeeding, supplements, tobacco and alcohol consumption,

c adjusted by mother´s education, state of residence, supplements, tobacco and alcohol consumption.

The results revealed that the consumption of allium vegetables during pregnancy was significantly negatively associated with AL for both medium and high consumption (OR=0.26, 95% CI 0.14-0.46), showing a trend in both models. Corn consumption was also negatively associated with AL (OR=0.32, 95% CI 0.16-0.64), with a *P*-trend for medium and high consumption levels. This was not observed for corn tortilla consumption, which was analyzed as a separate group. In the consumption of alcoholic beverages and avocado, something similar was observed for both models in the highest consumption group. Conversely, the consumption of high-fat dairy products during pregnancy was positively associated with AL at the medium and high consumption levels, with significance at the latter consumption level for both models (OR=2.37, 95% CI 1.30-4.34). In addition, a trend was found for the consumption of this food group (*P*<0.001). For other food groups studied, negative associations were found in model B, although they were not significant and were not observed to have trends in consumption during pregnancy, as in the high consumption level for citrus fruits (OR=0.56, 95% CI 0.32-0.98), foods rich in saturated fats (OR=0.38, 95% CI 0.20-0.69), cereals (OR=0.50, 95% CI 0.28-0.89), and Mexican dishes (OR=0.53, 95% CI 0.29-0.94). No association was found between consumption of topoisomerase II inhibitor foods during pregnancy and AL in this study group (**Table 3**).

## 4 Discussion

To the best of our knowledge, this is the first study to evaluate diet and the association with AL in children ≤24 months old in Mexico City. The results show a negative and positive association with maternal diet during pregnancy and AL in infants, with significant dose-response trends.

The negative association found between the consumption of allium vegetables during pregnancy and AL in their offspring is very interesting. Allium vegetables include garlic and onions, which are used worldwide in cooking for seasoning. There are numerous publications on the beneficial properties of these vegetables, but research in recent years has focused on the anti-cancer potential of these vegetables and their components. Allium vegetables contain water, carbohydrates, proteins, fiber, and fat, as well as essential amino acids, vitamins, and minerals (38). When garlic is minced or crushed, its membrane is ruptured and the S-allylcysteine sulfoxide (alliin) is enzymatically transformed into allicin by alliinase (39). Allicin is converted to mono-, di-, and tri-sulfide and other compounds, such as ajoene. Onions also contain mainly S-propenylcysteine sulfoxide and other sulfoxides, such as S-propylcysteine sulfoxide and S-methylcysteine sulfoxide (40). These organosulfur compounds are attributed with anticarcinogenic effects. The possible mechanisms of cancer prevention are suppression of mutagenesis, free radical scavenging, regulation of enzymatic activities, inhibition of protein folding in the endoplasmic reticulum, and inhibition of carcinogenic activity, such as proliferation, resistance to apoptosis, and evasion of immunosurveillance (41). Epidemiological studies provide various results on the cancer-preventive properties of allium vegetables; the strongest results are for the prevention of cancers of the gastrointestinal tract, including to some extent gastric, colorectal, and esophageal cancers (42). However, research studies are still underway to improve the method of assessing allium vegetable consumption and the amount needed to reduce the risk of cancer (43).

Similarly, the positive association observed between consumption of high-fat dairy foods during pregnancy and AL in infants is controversial. In general, consumption of milk and dairy products is recommended, especially during pregnancy, because they are an important source of protein, calcium, and B vitamins (thiamine, riboflavin, niacin, vitamin B6, and folate), also providing vitamin A, vitamin C, magnesium, and zinc (44). They also contain carbohydrates (lactose) and monounsaturated fats, both at low levels, and are one of the main sources of conjugated linoleic acid in the diet (45). The study of milk consumption worldwide and its relationship with cancer has yielded various results; some studies have shown that milk consumption has a protective effect for some types of cancer, such as colorectal cancer, breast cancer, ovarian cancer, bladder cancer, and prostate cancer (42). However, some studies have associated the consumption of milk and its derivatives as a risk factor for some types of cancer, such as prostate cancer, ovarian cancer, breast cancer, and bladder cancer (42–45). Milk has also been reported to contain estrogens, such as testosterone (46), which leads to increased cell division, activation of proto-oncogenes, and inactivation of tumor suppressor genes (47). Cow’s milk has also been reported to contain insulin-like growth factor I (IGF-I), which at high levels influences the regulation of proliferation, differentiation, apoptosis, and the development of neoplasms (48). Obesity leads to enhanced circulating levels of insulin, IGF-I, IGF-II and IGF-binding proteins (IGFBP-3), while reducing levels of the low molecular weight IGF-binding proteins (IGFBP-1, IGFBP-2). The effect of these changes is increased signaling through the insulin and IGF-I receptors, with resultant increased mitogenesis and decreased apoptosis, increasing the risk of carcinogenesis (49). In addition, other contaminants are likely to be present in milk, such as pesticides and veterinary drugs, and the process of homogenization or pasteurization by means of high temperatures potentiates the oxidation of fats, which can lead to the formation of free radicals and polymerized compounds that are carcinogenic (44). These mechanisms have not yet been studied in childhood AL, which would be a window of opportunity in this field.

The results in the present study cannot be compared to what has been published to date on maternal diet and AL development in children ≤24 months of age. Most epidemiological studies that have evaluated maternal diet and its association with AL in offspring have been in school-aged children and adolescents (8–15), because gathering an appropriate sample size in infants is difficult, options such as those proposed by CLIC of forming international groups to gather large sample sizes and look for associations (50).

Some of the findings in this study, such as the negative association with the consumption of fruits and vegetables, coincides with the findings reported by Jensen et al., who found that high consumption of fruits and vegetables by the mother during pregnancy decreases the risk of ALL (10), similar to what was also reported in the recent meta-analysis by Blanco-Lopez et al. (51). In contrast to the findings of Ross et al. (18), when we analyzed foods containing topoisomerase II inhibitors as a food group, among them coffee, we did not find an association with AL, similar to that reported by Milne et al. (52).

When interpreting the results of this study, the low response rate among cases and the random measurement error in the FFQ must be considered. Another limitation of this study are the collection on maternal feeding during pregnancy data after the diagnosis of AL in the cases and the interview in the controls, so there may be recall bias typical of case-control studies. However, the mothers who were interviewed did not know the main hypothesis of the study, and the methods used for data collection were the same between cases and controls; thus, any bias would be the same between cases and controls. Not knowing the study hypothesis prevented the mothers of the cases or controls from making a special effort to recall the consumption of a specific type of food that they believed was associated with AL. Similarly, not knowing the hypothesis prevented them from denying the consumption of any food that they believed could have caused leukemia. This reduced the risk of a differential error in the study. The questionnaire with sociodemographic variables included an open-ended question asking the mother why she believed her child had developed leukemia. This question helped us to identify how many mothers attributed their child’s disease to diet, and we identified 9 mothers who gave this answer and were therefore excluded from the analysis; this did not change the results (data not shown).The controls who participated in the study tended to have a higher socioeconomic status and education level than the cases, which could suggest selection bias. Importantly, participation of controls with better socioeconomic and education status has also been reported in population-based case-control studies, which have a higher willingness to participate (53).

Having controls with high socioeconomic status could produce an inverse association between, for example, the consumption of alcohol, foods rich in saturated fats, and Mexican dishes, which were foods that were identified as having negative associations for AL but without a significant trend. Thus, it would seem that the higher socioeconomic status in the control group favors the purchase and consumption of these foods. Nevertheless, in model B, we adjusted for maternal education and other variables, with no effect on the result.

Furthermore, we did not consider other variables as possible confounders, such as weight and height before pregnancy and weight gained during the trimesters of pregnancy in the mothers who participated in the study, or if it would have been possible to obtain data on body mass index (BMI) or fat mass index. This information would allow us to determine whether the mothers who participated were obese as, according to the literature, maternal obesity introduces genetic, hormonal, and biochemical changes in the intrauterine environment (54). Excess body weight is associated with increased levels of IGF-1 and IGF-2 and decreased levels of IGFBP-1 and IGFBP-2, which may result in increased mitogenesis and decreased apoptosis, increasing the risk of carcinogenesis (54, 55).

We also did not ask the mothers as to whether she had gestational diabetes during her pregnancy, which has been associated with the development of ALL in offspring (55). Insulin resistance may also play a role in the relationship between maternal obesity and the risk of ALL in offspring through inflammation (56). Inflammation, in turn, can promote leukemogenesis via several mechanisms, including through cytokines and chemokines, nuclear factor kappa B transcription factors, and signal transducer and activator of transcription 3 (STAT3) (57).

Epidemiological studies on diet or other environmental factors in infants are absolutely necessary for the study of leukemogenesis because they have a short period of exposure (before and during pregnancy) for the manifestation of the disease (58). The usefulness of identifying food groups or specific foods associated with AL in children allows the planning of prevention strategies to promote an increase or decrease in their consumption in pregnant women or women planning to become pregnant with the purpose of adding to the health recommendations in this reproductive stage with implications not only for AL in infants, but also for the development of AL in children and adolescents in the future.

### 4.1 Conclusion

In conclusion, the results of this study suggest that maternal intake of allium vegetables, specifically garlic, during pregnancy is inversely associated with the development of AL in children ≤24 months of age. On the other hand, consumption of high-fat dairy products, particularly milk, Oaxaca cheese, Manchego cheese, and fresh cheese, is positively associated with AL in children ≤24 months of age. Further research with some methodological considerations is required to corroborate the findings of this study.

## Data availability statement

The original contributions presented in the study are included in the article/supplementary material. Further inquiries can be directed to the corresponding authors.

## Ethics statement

The studies involving humans were approved by CNIC Instituto Mexicano del Seguro Social 2010-785-064. The studies were conducted in accordance with the local legislation and institutional requirements. Written informed consent for participation in this study was provided by the participants’ legal guardians/next of kin.

## Author contributions

MP-S, JM-A, and AF-G contributed to conception and design of the study. JF-L and DD-R organized the database. MP-S, MF-G, LL-C, and JN-E performed the statistical analysis. MP-S wrote the first draft of the manuscript. MF-G wrote sections of the manuscript. LL-C and JM-A reviewed it critically for important intellectual content. NN-V, AM-S, EJ-H, JM-T, NL-S, JP-G, BC-H, LM-P, RA-S, LG-L, HP-L, PR-Z, AC-E, ML-C, SM-S, JR-G, JG-K, JF-B, FM-L, MR-V, DT-V, KM-R, FM-R, LG-C, PS-L, VB-M, MM-R, HR-V, and SJ-M contributed administratively, technically, or financially. All authors contributed to the article and approved the submitted version.

## Figures and Tables

**Figure 1:**
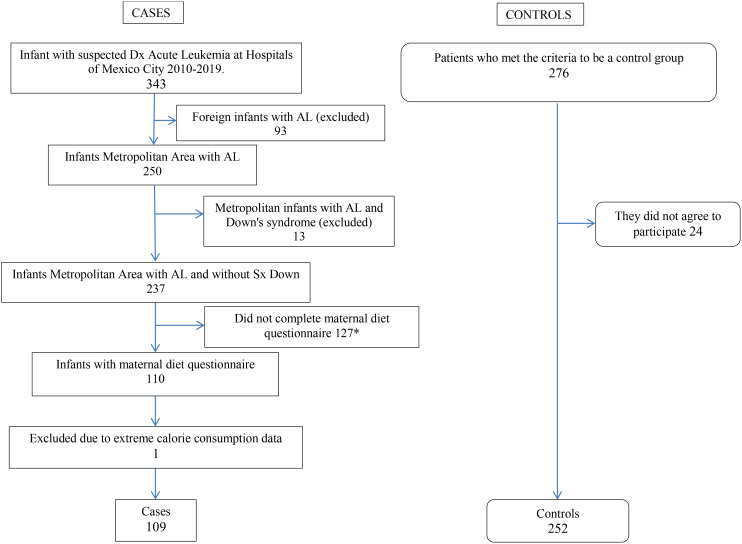
Diagram of the search and selection of the case and controls infant’s ≤ 24 months of age attended in public hospitals in Mexico City and State of Mexico 2010-2019. *11 patients were diagnosed in 2010 when the instrument was not yet available and could not be recovered, 11 patients whose mothers did not agree to participate, 10 patients who were treated in a institution that did not participate in the study, 55 patients who died within 2 months of diagnosis and could not be interviewed, 2 patients who were lost due to the COVID-19 pandemic, and 38 patients who were lost to follow-up and were not administered the material diet questionnaire.

**Figure:**
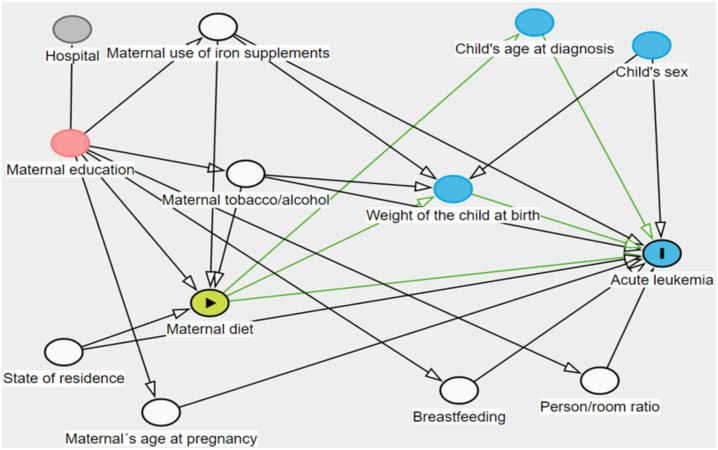
Directed acyclic graph of maternal diet intake and acute leukemia. White circles= adjusting variables; red circles= ancestor of exposure and outcome that does not need to be adjusted for; blue circle other than acute leukemia circle= ancestor of outcome. Green lines correspond to direct and indirect causal paths between maternal diet and Acute Leukemia, the latter are mediated by child’s age at diagnosis and weight of the child at birth.

## References

[1] Parkin DM Stiller CA Draper GJ Bieber CA. The international incidence of childhood cancer. Int J Cancer. (1988) 42(4):511–20. doi: 10.1002/ijc.2910420408 3170025

[2] Fajardo Gutiérrez A Mejía Aranguré M Gómez Delgado A Mendoza Sánchez H Garduño Espinosa J Martínez García M del C. Epidemiología de las neoplasias malignas en niños residentes del Distrito Federal (1982-1991). Boletín Médico del Hosp Infant México (Ed española) (1995) 52(9):507–16.

[3] Ross JA Davies SM Potter JD Robison LL. Epidemiology of childhood leukemia, with a focus on infants. Epidemiol Rev (1994) 16(2):243–72. doi: 10.1093/oxfordjournals.epirev.a036153 7713179

[4] Sam TN Kersey JH Linabery AM Johnson KJ Heerema NA Hilden JM. MLL gene rearrangements in infant leukemia vary with age at diagnosis and selected demographic factors: a children’s oncology group (COG) study. Pediatr Blood Cancer. (2012) 58(6):836–9. doi: 10.1002/pbc.23274 PMC3208122 21800415

[5] Johnson KJ Roesler MA Linabery AM Hilden JM Davies SM Ross JA. Infant leukemia and congenital abnormalities: a children’s oncology group study. Pediatr Blood Cancer. (2010) 55(1):95–9. doi: 10.1002/pbc.22495 PMC2904947 20486175

[6] Greaves M. Childhood leukaemia. BMJ (2002) 324(7332):283–7. doi: 10.1136/bmj.324.7332.283 PMC1122200 11823363

[7] Borge TC Aase H Brantsæter AL Biele G. The importance of maternal diet quality during pregnancy on cognitive and behavioural outcomes in children: a systematic review and meta-analysis. BMJ Open (2017) 7(9). doi: 10.1136/bmjopen-2017-016777 PMC5623570 28947450

[8] Thompson JR Gerald PF Willoughby ML Armstrong BK. Maternal folate supplementation in pregnancy and protection against acute lymphoblastic leukaemia in childhood: a case-control study. Lancet (2001) 358(9297):1935–40. doi: 10.1016/S0140-6736(01)06959-8 11747917

[9] Milne E Royle JA Miller M Bower C de Klerk NH Bailey HD. Maternal folate and other vitamin supplementation during pregnancy and risk of acute lymphoblastic leukemia in the offspring. Int J Cancer. (2010) 126(11):2690–9. doi: 10.1002/ijc.24969 19839053

[10] Jensen CD Block G Buffler P Ma X Selvin S Month S. Maternal dietary risk factors in childhood acute lymphoblastic leukemia (United states). Cancer Causes Control. (2004) 15(6):559–70. doi: 10.1023/B:CACO.0000036161.98734.17 15280635

[11] Sarasua S Savitz DA. Cured and broiled meat consumption in relation to childhood cancer: Denver, colorado (United states). Cancer Causes Control. (1994) 5(2):141–8. doi: 10.1007/BF01830260 8167261

[12] Bonaventure A Rudant J Goujon-Bellec S Orsi L Leverger G Baruchel A. Childhood acute leukemia, maternal beverage intake during pregnancy, and metabolic polymorphisms. Cancer Causes Control. (2013) 24(4):783–93. doi: 10.1007/s10552-013-0161-9 23404349

[13] Blot WJ Henderson BE Boice JD. Childhood cancer in relation to cured meat intake: review of the epidemiological evidence. Nutr Cancer. (1999) 34(1):111–8. doi: 10.1207/S15327914NC340115 10453449

[14] Peters JM Preston-Martin S London SJ Bowthan JD Buckley JD Thomas DC. Processed meats and risk of childhood leukemia (California, USA). Cancer Causes Control. (1994) 5(2):195–202. doi: 10.1007/BF01830266 8167267

[15] Liu CY Hsu YH Wu MT Pan PC Ho CK Su L. Cured meat, vegetables, and bean-curd foods in relation to childhood acute leukemia risk: A population based case-control study. BMC Cancer. (2009) 9:15. doi: 10.1186/1471-2407-9-15 19144145 PMC2653540

[16] Ross JA. Environmental and genetic susceptibility to MLL-defined infant leukemia. J Natl Cancer Inst Monogr (2008) 39):83–6. doi: 10.1093/jncimonographs/lgn007 18648010

[17] Ross JA Potter JD Robison LL. COMMENTARY infant leukemia, topoisomerase II inhibitors, and the MLL gene. (1994). doi: 10.1093/jnci/86.22.1678 7966394

[18] Ross JA Potter JD Reaman GH Pendergrass TW Robison LL. Maternal exposure to potential inhibitors of DNA topoisomerase II and infant leukemia (United states): a report from the children’s cancer group. Cancer Causes Control. (1996) 7(6):581–90. doi: 10.1007/BF00051700 8932918

[19] Spector LG Xie Y Robison LL Heerema NA Hilden JM Lange B. Maternal diet and infant leukemia: the DNA topoisomerase II inhibitor hypothesis: a report from the children’s oncology group. Cancer Epidemiol Biomarkers Prev (2005) 14(3):651–5. doi: 10.1158/1055-9965.EPI-04-0602 15767345

[20] Petridou E Ntouvelis E Dessypris N Terzidis A Trichopoulos D. Maternal diet and acute lymphoblastic leukemia in young children. Cancer Epidemiol Biomarkers Prev (2005) 14(8):1935–9. doi: 10.1158/1055-9965.EPI-05-0090 16103440

[21] Mejía-Aranguré JM Fajardo-Gutiérrez A Bernáldez-Ríos R Farfán-Canto JM Ortíz-Hernández A M-GM Mejía-Aranguré JM. Incidence trends of acute leukemia among the children of mexico city: 1982-1991. Arch Med Res (1996) 27(2):223–7. 8696068

[22] González-Block MA Reyes-Morales H Cahuana-Hurtado L Balandran A ME. Mexico: health system review. In: Policies EO on HS and, editor, 1st ed. World Health Organization. Regional Office for Europe: World Health Organization (2020). p. 222.

[23] Hernández-Avila M Romieu I Parra S Hernández-Avila J Madrigal H Willett W. Validity and reproducibility of a food frequency questionnaire to assess dietary intake of women living in mexico city. Salud Publica Mex. (1998) 40(2):133–40. doi: 10.1590/S0036-36341998000200005 9617194

[24] Willett WC Sampson L Stampfer MJ Rosner B Bain C Witschi J. Reproducibility and validity of a semiquantitative food frequency questionary. Am J Epidemiol. (1985) 122(1):51–65. doi: 10.1093/oxfordjournals.aje.a114086 4014201

[25] USDA-ARS. Composition of foods raw, processed, prepared USDA national nutrient database for standard reference, release 20. 20th ed. Center BHNR, editor. Maryland: Nutrient Data Laboratory (2007) p. 1–49.

[26] Muñoz de Chávez M Chávez A Roldan JA Ledesma JA Mendoza E Pérez-Gil RF. Tablas de valor nutritivo de los alimentos de mayor consumo en méxico. 1a ed. México: Pax (1996). p. 330.

[27] Stelmach-Mardas M Kleiser C Uzhova I Penalvo JL La Torre G Palys W. Seasonality of food groups and total energy intake: a systematic review and meta-analysis. Eur J Clin Nutr (2016) 70(6):700–8. doi: 10.1038/ejcn.2015.224 26757837

[28] National Institute of Diabetes and Digestive and kidney Diseases. Food portions: Choosing just enough for you - NIDDK [Internet] . Available at: https://www.niddk.nih.gov/health-information/weight-management/just-enough-food-portions.

[29] Tijerina Sáenz A Ramírez López E Meneses Valderrama VM MGE. Intakes of energy and macronutrients in pregnant women in the northeast of mexico. Arch latinoaméricanos Nutr (2014) 64(3):1–14. 26137793

[30] Dockerty JD Draper G Vincent T Rowan SD Bunch KJ. Case-control study of parental age, parity and socioeconomic level in relation to childhood cancers. Int J Epidemiol. (2001) 30(6):1428–37. doi: 10.1093/ije/30.6.1428 11821358

[31] Poole C Greenland S Luetters C Kelsey JL Mezei G. Socioeconomic status and childhood leukaemia: a review. Int J Epidemiol. (2006) 35(2):370–84. doi: 10.1093/ije/dyi248 16308412

[32] Jiménez-Hernández E Fajardo-Gutiérrez A Núñez-Enriquez JC Martín-Trejo J Espinoza-Hernández LE Flores-Lujano J. A greater birthweight increases the risk of acute leukemias in mexican children-experience from the mexican interinstitutional group for the identification of the causes of childhood leukemia (MIGICCL). Cancer Med (2018). 10.1002/cam4.1414 PMC5911591 29533016

[33] Marcotte EL Thomopoulos TP Infante-Rivard C Clavel J Petridou ET Schüz J. Caesarean delivery and risk of childhood leukaemia: a pooled analysis from the childhood leukemia international consortium (CLIC). Lancet Haematol (2016) 3(4):e176–85. doi: 10.1016/S2352-3026(16)00002-8 PMC5283076 27063976

[34] Petridou ET Georgakis MK Erdmann F Ma X Heck JE Auvinen A. Advanced parental age as risk factor for childhood acute lymphoblastic leukemia: Results from studies of the childhood leukemia international consortium. Eur J Epidemiol. (2018) 33(10):965–76. doi: 10.1007/s10654-018-0402-z PMC6384148 29761423

[35] Núñez-Enríquez JC Correa-Correa V Flores-Lujano J Pérez-Saldivar ML Jiménez-Hernández E Martín-Trejo JA. Extremely low-frequency magnetic fields and the risk of childhood b-lineage acute lymphoblastic leukemia in a city with high incidence of leukemia and elevated exposure to ELF magnetic fields. Bioelectromagnetics (2020) 41(8):581–97. doi: 10.1002/bem.22295 32965755

[36] Howards PP Schisterman EF Poole C Kaufman JS Weinberg CR. “Toward a clearer definition of confounding” revisited with directed acyclic graphs. Am J Epidemiol. (2012) 176(6):506–11. doi: 10.1093/aje/kws127 PMC3530354 22904203

[37] Rothman KJ Greenland S. Modern epidemiology. 2nd ed. Philadelphia, USA: Lippincott-Raven Publisher (1998).

[38] Wan Q Li N Du L Zhao R Yi M Xu Q. Allium vegetable consumption and health: An umbrella review of meta-analyses of multiple health outcomes. Food Sci Nutr (2019) 7(8):2451–70. doi: 10.1002/fsn3.1117 PMC6694434 31428334

[39] Nicastro HL Ross SA Milner JA. Garlic and onions: their cancer prevention properties. Cancer Prev Res (Phila). (2015) 8(3):181–9. doi: 10.1158/1940-6207.CAPR-14-0172 PMC4366009 25586902

[40] Nohara T Fujiwara Y El-Aasr M Ikeda T Ono M Nakano D. Antitumor allium sulfides. Chem Pharm Bull (Tokyo). (2017) 65(3):209–17. doi: 10.1248/cpb.c16-00844 28250342

[41] Bianchini F Vainio H. Allium vegetables and organosulfur compounds: do they help prevent cancer? Environ Health Perspect (2001) 109(9):893–902. doi: 10.1289/ehp.01109893 11673117 PMC1240438

[42] Papadimitriou N Markozannes G Kanellopoulou A Critselis E Alhardan S Karafousia V. An umbrella review of the evidence associating diet and cancer risk at 11 anatomical sites. Nat Commun (2021) 12(1):1–10. doi: 10.1038/s41467-021-24861-8 34321471 PMC8319326

[43] Zhang Y Liu X Ruan J Zhuang X Zhang X Li Z. Phytochemicals of garlic: Promising candidates for cancer therapy. BioMed Pharmacother. (2020) 123(109730). doi: 10.1016/j.biopha.2019.109730 31877551

[44] Davoodi H Esmaeili S Mortazavian AM. Effects of milk and milk products consumption on cancer: A review. Compr Rev Food Sci Food Saf. (2013) 12(3):249–64. doi: 10.1111/1541-4337.12011

[45] Miller G Jarvis J McBean L. Handbook of dairy foods and nutrition, second edition. In: Handbook of dairy foods and nutrition, second edition, 3rd ed. Boca Raton Florida: CRC Press Taylor and Francis Group (1999). p. 401.

[46] Dorgan JF Judd JT Longcope C Brown C Schatzkin A Clevidence BA. Effects of dietary fat and fiber on plasma and urine androgens and estrogens in men: a controlled feeding study. Am J Clin Nutr (1996) 64(6):850–5. doi: 10.1093/ajcn/64.6.850 8942407

[47] Ross RK Henderson BE. Do diet and androgens alter prostate cancer risk via a common etiologic pathway? J Natl Cancer Inst (1994) 86(4):252–5. doi: 10.1093/jnci/86.4.252 8158675

[48] Yu H Rohan T. Role of the insulin-like growth factor family in cancer development and progression. J Natl Cancer Inst (2000) 92(18):1472–89. doi: 10.1093/jnci/92.18.1472 10995803

[49] Simmen FA Simmen RCM. The maternal womb: a novel target for cancer prevention in the era of the obesity pandemic? Eur J Cancer Prev (2011) 20(6):539. doi: 10.1097/CEJ.0b013e328348fc21 21701386 PMC3184194

[50] Metayer C Milne E Clavel J Infante-Rivard C Petridou E Taylor M. The childhood leukemia international consortium. Cancer Epidemiol. (2013) 37(3):336–47. doi: 10.1016/j.canep.2012.12.011 PMC3652629 23403126

[51] Blanco-Lopez J Iguacel I Pisanu S Almeida CCB Steliarova-Foucher E Sierens C. Role of maternal diet in the risk of childhood acute leukemia: A systematic review and meta-analysis. Int J Environ Res Public Health (2023) 20(7). doi: 10.3390/ijerph20075428 PMC10093835 37048042

[52] Milne E Greenop KR Petridou E Bailey HD Orsi L Kang AY. Maternal consumption of coffee and tea during pregnancy and risk of childhood ALL: a pooled analysis from the childhood leukemia international consortium. Cancer Causes Control. (2018) 29(6):539–50. doi: 10.1007/s10552-018-1024-1 29600472

[53] Mazloum M Bailey HD Heiden T Armstrong BK De Klerk N Milne E. Participation in population-based case-control studies: does the observed decline vary by socio-economic status? Paediatr Perinat Epidemiol (2012) 26(3):276–9. doi: 10.1111/j.1365-3016.2011.01253.x 22471687

[54] Marley AR Ryder JR Turcotte LM Spector LG. Maternal obesity and acute lymphoblastic leukemia risk in offspring: A summary of trends, epidemiological evidence, and possible biological mechanisms. Leuk Res (2022) 121. doi: 10.1016/j.leukres.2022.106924 35939888

[55] Søegaard SH Rostgaard K Kamper-Jørgensen M Schmiegelow K Hjalgrim H. Maternal diabetes and risk of childhood acute lymphoblastic leukaemia in the offspring. Br J Cancer. (2018) 118(1):117–20. doi: 10.1038/bjc.2017.351 PMC5765219 28972964

[56] Shoelson SE Herrero L Naaz A. Obesity, inflammation, and insulin resistance. Gastroenterology (2007) 132(6):2169–80. doi: 10.1053/j.gastro.2007.03.059 17498510

[57] Krawczyk J O’Dwyer M Swords R Freeman C Giles FJ. The role of inflammation in leukaemia. Adv Exp Med Biol (2014) 816:335–60. doi: 10.1007/978-3-0348-0837-8_13 24818729

[58] Emerenciano M Koifman S Pombo-de-Oliveira MS. Acute leukemia in early childhood. Braz J Med Biol Res (2007) 40(6):749–60. doi: 10.1590/S0100-879X2007000600002 17581672

